# Piezoelectric Wafer Active Sensor Transducers for Acoustic Emission Applications

**DOI:** 10.3390/s23167103

**Published:** 2023-08-11

**Authors:** Connor Griffin, Victor Giurgiutiu

**Affiliations:** Department of Mechanical Engineering, University of South Carolina, Columbia, SC 29208, USA

**Keywords:** piezoelectric wafer active sensors, structural health monitoring

## Abstract

Piezoelectric materials are defined by their ability to display a charge across their surface in response to mechanical strain, making them great for use in sensing applications. Such applications include pressure sensors, medical devices, energy harvesting and structural health monitoring (SHM). SHM describes the process of using a systematic approach to identify damage in engineering infrastructure. A method of SHM that uses piezoelectric wafers connected directly to the structure has become increasingly popular. An investigation of a novel pitch-catch method of determining instrumentation quality of piezoelectric wafer active sensors (PWASs) used in SHM was conducted as well as an investigation into the effects of defects in piezoelectric sensors and sensor bonding on the sensor response. This pitch-catch method was able to verify defect-less instrumentation quality of pristinely bonded PWASs. Additionally, the pitch-catch method was compared with the electromechanical impedance method in determining defects in piezoelectric sensor instrumentation. Using the pitch-catch method, it was found that defective instrumentation resulted in decreasing amplitude of received and transmitted signals as well as changes in the frequency spectrums of the signals, such as the elimination of high frequency peaks in those with defects in the bonding layer and an increased amplitude of around 600 kHz for a broken PWAS. The electromechanical impedance method concluded that bonding layer defects increase the primary frequency peak’s amplitude and cause a downward frequency shift in both the primary and secondary frequency peaks in the impedance spectrum, while a broken sensor has the primary peak amplitude reduced while shifting upward and nearly eliminating the secondary peak.

## 1. Introduction

For piezoelectric materials in sensing applications, the direct piezoelectric effect is utilized, where there is a conversion of mechanical energy to electrical energy. Mechanical strain applied to a piezoelectric sensor results in the polarization of the sensor, causing a difference in electric potential across the sensor that can be measured. Applications of piezoelectric sensors and the direct piezoelectric effect include industries such as the automotive industry, medical devices, Internet of Things (IoT) and energy harvesters [1]. Another application of piezoelectric sensors is in the field of SHM.

### 1.1. PWAS for SHM Applications

SHM describes the process of implementing a strategy for identifying damage in aerospace, civil and mechanical engineering infrastructure. SHM uses systematically spaced measurements, and the analysis of these measurements related to damage, to determine the state of the system [2]. SHM uses sensors attached to mechanical parts to collect information on the performance and health of the specimen. Monitoring the structural state allows for design feedback, performance enhancement and on-demand condition-based maintenance. A SHM system can consist of sensors, clusters and electronics working in conjunction with data processing and communication systems. A method of SHM that uses PWASs connected directly to the structure has become increasingly popular [3].

**Figure 1:**
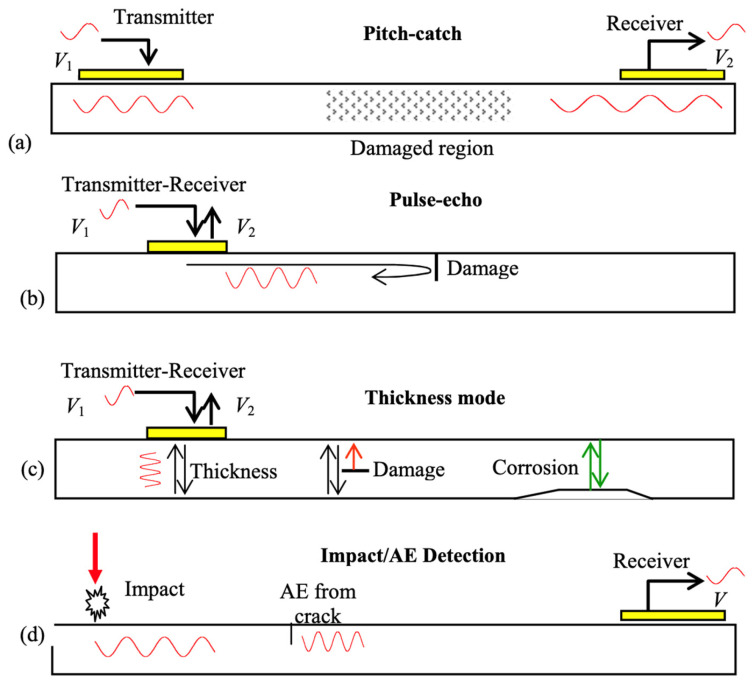
Possible uses of piezoelectric wafer active sensors (PWASs) in SHM applications; (**a**) pitch-catch; (**b**) pulse-echo; (**c**) thickness mode; (**d**) impact/AE detection [3].

PWASs are cheap transducers that act based upon piezoelectric principles. PWASs are similar in price to a conventional resistance strain gauge, but with greater performance as they are capable of actively examining the structure at will. PWASs are capable of being used in high-frequency applications exceeding hundreds of kHz, as well as being used as actuators and sensors of elastic waves and vibration. Through the analysis of wave propagation and vibration, the existence, location and severity of structural damage can be determined. A PWAS bonded to the surface of a structure couples in plane strain with the strain on the surface of the structure [4]. These PWASs have become popular due to their versatility in use and monitoring methods, including pitch-catch, pulse-echo and phased array, the electromechanical impedance method, as well as sensing of acoustic emissions and impact detection [3]. Examples of pitch-catch, pulse-echo, thickness mode and AE detection PWAS modes for SHM are depicted in Figure 1. Though PWAS transducers have both transmitting and receiving capabilities [4], the focus of the present work is to prove their capabilities as receivers of AE wave signals. Previously reported comparative studies [5,6] have established that these relatively inexpensive sensors have as good a performance as the conventional but more expensive AE sensors R15a, PICO and S9225 [7,8].

The durability and consistency of a PWAS transducer is of paramount importance. It is essential that, during SHM applications, the PWAS transducers signals are consistent and truly representing the state of the structure on which they are mounted. The PWAS signals should not be affected by the degradation of the adhesive bond between the PWAS and the structure, or by the degradation of the PWAS itself. Hence, the durability of PWAS instrumentation must be understood to ensure that the instrumentation maintains its integrity throughout the SHM process.

The durability of PWASs has been studied [9] and their applicability for use in harsh environments has been verified through material characterization, showing that there is no noteworthy change in the microstructure of piezoelectric sensors after exposure to high temperatures and radiation, thus solidifying their potential for application in the SHM of structures in demanding environments such as those associated with aerospace. PWASs can be used for sensing in SHM in two modes: active sensing and passive sensing. Passive sensing using PWASs monitors the structure passively, only during the selected time period, while PWASs used in active sensing analyzes the structure for damage by exciting the structure and then monitoring the response. PWAS use for active sensing includes detection of far-field damage using pulse-echo, pitch-catch and phased array methods, and the detection of near-field damage using the electromechanical impedance method. PWASs are used in the passive sensing of acoustic emissions from crack advancement as well as impact detection. Recent progress in the LAMSS research group using PWASs for SHM includes the active sensing of crack and corrosion damage in aluminum plates, the active sensing of delamination in composite structures through the analysis of lamb waves and the passive sensing of acoustic emissions during fatigue crack growth [9].

### 1.2. Previous Work on PWAS Instrumentation Quality Checks

The quality of PWAS instrumentation in SHM is of particular importance because incorrect measurements resulting from faulty sensors or sensor bonding can lead to distorted signals and impair the ability to monitor the structure. Regarding the quality of the sensor, previous research has been done into the analysis of the quality of the PWAS bond to the structure as well as its effect on the sensing capabilities of the PWAS [10,11]. Much of this research is associated with the electromechanical impedance spectrum (EMIS) of a free PWAS compared to that of a bonded one. Investigations have also been conducted into the change in the EMIS of a bonded PWAS as its quality, environment and bonding conditions change. Changes in the EMIS of a PWAS can indicate damage and reduced sensing capabilities or complete detachment from the structure. Lin et al. [10] used changes in the real part of the EMIS to monitor the durability, survivability and bonding state of PWASs exposed to both high and low temperatures, outdoor exposure, temperature cycles, submersion and overfatigue testing. T Wandowski et al. [12] discussed the use of time-domain terahertz spectroscopy for the determination of piezoelectric sensor bond quality. Time-domain terahertz spectroscopy was used to determine the distribution of the bond layer between the piezoelectric sensor and the structure, while the electromechanical impedance method was utilized to verify the results of the time-domain terahertz spectroscopy. Results for the THz spectroscopy and electromechanical impedance method show that the real part of impedance spectrum when the sensor is bonded to the structure displays peaks related to the resonant frequencies of the monitored structure, and that when a PWAS is bonded improperly or becomes partially disbonded the frequencies peak then shift towards or become the resonant frequencies of the piezoelectric sensor itself. The electromechanical impedance method is useful in determining PWAS bond quality, as long as the resonance peaks of the free PWASs are known.

Park et al. [13] investigated the determination of defective piezoelectric sensor bonding through use of the electromechanical impedance method, as well as the effects of defects in sensor bonding using wave propagation. Their analysis centers on the imaginary part of the admittance. Their findings include that piezoelectric sensors bonded to a specimen experience a decrease in the slope of the imaginary part of their admittance spectrum due to the bonding layer when compared to that measured for a free sensor. Through this analysis, an increased slope of the imaginary part of the admittance spectrum compared to that of a perfectly bonded sensor can be used to identify sensors with bonding defects. In their analysis, defective bonding identified through the imaginary part of the admittance of a sensor resulted in alterations in the amplitude, phase and shape of propagating lamb waves. In addition to their investigation into the analysis of the imaginary part of admittance, the real part of the impedance spectrum of sensors was analyzed. It should be noted that with changing bonding conditions, the values and amplitudes of resonant frequencies change. Saravanan and Chauhan [14] used conductance and susceptance to analyze the debonding and breaking of piezoelectric patches used in SHM. Through both theoretical and experimental methods, they determined the admittance signature for free piezoelectric patches, perfectly bonded patches, disbonded patches and broken patch sensors. Results show that for sensor debonding, a decrease in the susceptance slope occurs, although only by a small percentage. Both experimental and simulation results show that with sensor debonding there is an upward shift in the conductance signature, with no change in resonant frequency and sensor degradation or breaking corresponding with a downward shift in resonant frequency.

Mueller et al. [15] analyzed the generated wave field and electromechanical impedance spectrum of piezoelectric transducers at varying bonding conditions. The bonding conditions studied included fully bonded, 20% disbonded, 50% disbonded and 80% disbonded. Their analysis of the wave field produced by partially disbonded transducers actuating at various frequencies indicated that the effect of debonding on the wave generation of piezoelectric sensors is dependent on the location of the debonding relative to the electrode, as well as the frequency of the actuating PWAS. In some instances, this debonding produced an increase in amplitude of the wavefield due to energy trapping and localization, while in others it produced a decrease. In their analysis of the susceptance of disbonded sensors, it was found that there are additional resonance peaks in disbonded PWASs as well as a shift in resonance and an increase in slope of the low frequency range. Mueller and Fritzen [11] discussed the effect of PWAS faults, as well as PWAS degradation caused by dimethylformamide on wave propagation and EMIS in simple PWASs as well as embedded PWASs. Their conclusions included that different PWAS faults have a significant effect on the wave propagation of the sensors. It was found that breaking of a PWAS leads to a shift in the resonance peak of the susceptance, while spalling leads to a shift in the susceptance slope. For an embedded PWAS, the only changes are observed in the frequency range higher than PWAS resonance. PWAS bond degradation led to a shift in the resonance toward lower frequencies for a simple transducer, while for an embedded transducer this effect is less pronounced. Current methods of determining PWAS instrumentation quality are mainly concerned with measurement of the EMIS of a free sensor, or a sensor bonded to the structure. In the following sections, a new method of PWAS instrumentation inspection is introduced that is centered upon analysis of wave propagation through the structure and measurement of signals received by PWAS.

### 1.3. Focus of This Journal Article

This journal article focuses on improving the methods for assessing the quality of PWAS instrumentation for acoustic emission applications. The existing approaches for assessing the quality of PWAS instrumentation primarily focus on evaluating the EMIS of a standalone sensor or sensor bonded to a structure. Using this method, the sensing and receiving capabilities are not directly measured and instead the resonant frequencies of the sensors are the subject of investigation.

In our approach, we have pursued two avenues for assessing PWAS instrumentation quality. One avenue was to study the reciprocity of PWASs acting alternatively as receiver and transmitter of guided ultrasonic waves. For a symmetric arrangement of PWAS transducers, the reciprocity approach allows us to identify if problems of PWAS placement may exist due to human error. Using a pitch-catch approach, it became possible to determine sensor quality based on the signals a sensor receives and transmits. This method allows for the analysis of the sensor quality using the same data collected during an acoustic emission as well as allowing for not only the investigation of sensor instrumentation quality but also an investigation into the effects of instrumentation defects on the acoustic emission signal.

The other avenue pursued in our study relates to the quality of the sensor itself and of its attachment to the structural substrate. In this case, we used the EMIS approach. Although the EMIS approach was also used previously by other investigators, we enhanced this approach by creating carefully conducted test cases to distinguish between various low-quality situations (e.g., a poor sensor bond vs. a partially broken sensor) and see how these situations could be identify in the EMIS spectrum.

We believe that our multi-pronged approach described in this article would be quite useful in practical applications that are concerned with PWAS instrumentation quality and integrity.

## 2. Experimental Setup and Procedures: Discussion of Experimental Setup and Testing Performed

**Figure 2:**
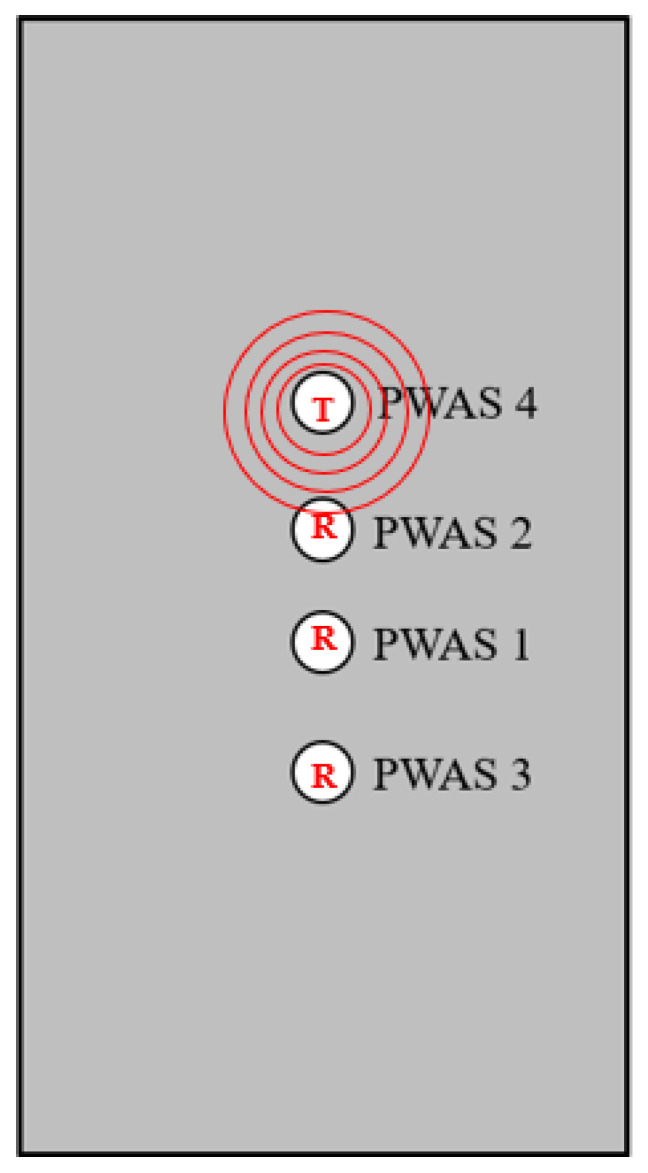
Diagram of pitch-catch experimentation where T represents the transmitting PWAS and R represents the receiving PWAS.

When instrumenting a specimen with a PWAS for SHM, some important factors are the quality of the sensor bonding, the accurate and precise placement of the sensors on the specimen and the integrity and similarity of the sensors. To inspect the piezoelectric instrumentation, a pitch-catch method was introduced. The pitch-catch method consists of one PWAS transmitting a signal and the other PWASs receiving the signal, then comparing the PWAS response between the receiving sensors. This pitch-catch method can be performed in a round robin fashion, with each PWAS rotating as transmitter and receiver. With defect-less sensor instrumentation, waveforms received at PWASs should be the same as at sensors that are the same distance from the transmitter. A diagram of the PWAS pitch-catch experiment is shown in Figure 2, where T represents the transmitting sensor and R represents the receiving sensors.

Using the numbering convention pictured in Figure 2, it is expected that due to the symmetry of instrumentation, the waveforms received at PWASs 4, 2 and 1 when PWAS 3 is transmitting should be the similar to the waveforms received at PWASs 3, 1 and 2 when PWAS 4 is transmitting. This would be the same case at symmetric PWASs while PWASs 1 and 2 are the transmitting PWASs.

**Figure 3:**
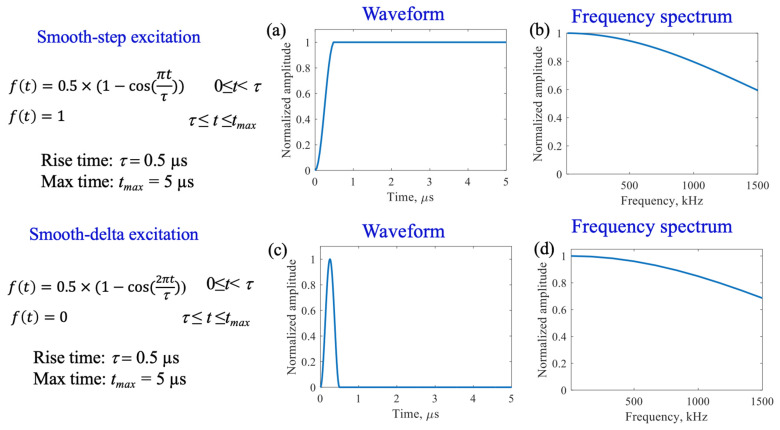
Equations, waveforms and frequency spectrum for excitation signals. (**a**) Smooth−step waveform; (**b**) Smooth−step frequency spectrum; (**c**) Smooth−delta waveform; (**d**) Smooth−delta frequency spectrum.

In order to check our instrumentation in this round robin fashion, two actuation functions were used for the transmitting PWAS. One function was a smooth-step function associated with fatigue crack growth acoustic emissions in the finite element modeling of fatigue cracks, while the other function was a smooth-delta function associated with fatigue crack rubbing and clapping [16,17,18]. Each function had a rise time of 0.5 microseconds, with each function’s equations, waveforms and frequency spectrums being shown in Figure 3. The frequency spectrums show the ability of the numerically simulated step and delta functions to excite high-frequency components with only a gradual decrease as the frequency increases. The spectra are similar, but the step spectrum decreases faster than the delta spectrum. Please note that, in the step spectrum, we eliminated the DC component for f = 0 Hz, which is not of interest to our study.

**Figure 4:**
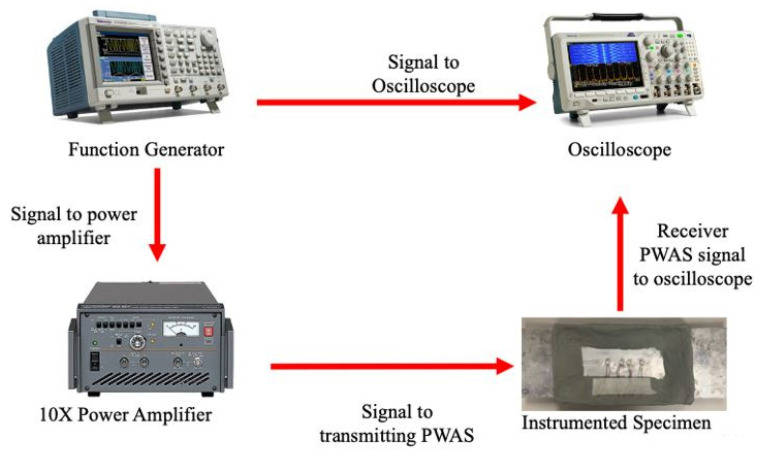
Experimental setup for pitch-catch experimentation.

For the actuation of PWASs, as well as the collection of received signals from PWASs, the experimental setup shown in Figure 4 was used. This setup consisted of a function generator that communicates the actuation signal, either the smooth-step or smooth-delta function, to the connected oscilloscope as well as the 10× power amplifier. From the power amplifier, the actuation signal is then sent to the transmitting PWAS. The signal from each sensing PWAS is then sent to the oscilloscope to be collected. Collection of signals from the oscilloscope lends the actuation function sent to the transmitter and the signals received at each of the receiving sensors.

Pitch-catch experimentation was first performed on a pristine specimen instrumented with 4 PWAS, as previously discussed and shown in Figure 2. Within this PWAS placement, the distance from the horizontal line of symmetry of sensor placement to PWAS 1 and PWAS 2 was 5 mm, and the distance from this line of symmetry to each of PWASs 3 and 4 was 25 mm. The specimen instrumented was a 12 in. × 4 in. Al-2024 coupon, with a 7 mm diameter, 0.5 mm thickness, circular PWAS bonded to its surface using M bond AE 15 adhesive. A clay, non-reflective boundary was applied along the edges of the coupon to eliminate free vibration of the plate and prevent the reflection of lamb waves transmitted by the sensors across the plate. Currently, this PWAS instrumentation setup is one that was being utilized to collect acoustic emissions generated during fatigue crack growth. In the case of acoustic emission collection, the fatigue crack was grown between PWASs 1 and 2, allowing for the collection of the acoustic emissions as well as verification the acoustic emission has occurred from the fatigue crack and not outside sources.

**Figure 5:**
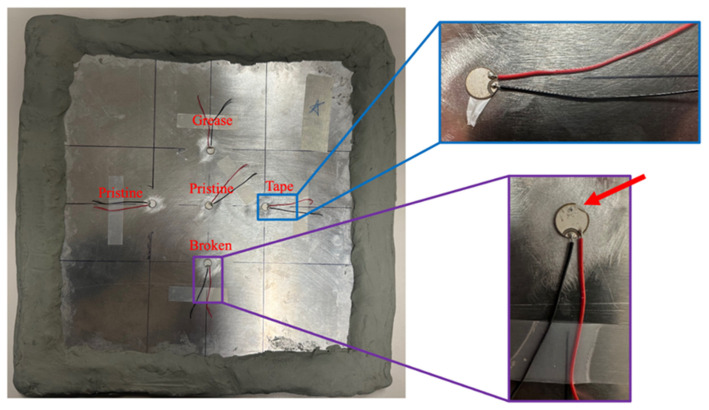
Specimen instrumented with two pristine PWASs, one broken PWAS, one PWAS with tape beneath the bonding layer and one PWAS with grease beneath the bonding layer.

Additionally, for investigation into the effects of defects on the PWAS response, a specimen was instrumented with defective instrumentation and pitch-catch testing was performed. The specimen was a 12 in. × 12 in. Al-2024 plate, again instrumented with 7 mm diameter, 0.5 mm thick, circular PWAS. M bond AE 15 adhesive was again used to fix the PWAS to the specimen and the same clay non-reflective boundary was applied. The PWASs selected for instrumentation on both specimens possessed similar capacitance and EMIS values to best maintain signal similarity between sensors. This specimen was instrumented with five PWASs, two pristine, one broken, one with a small dollop of grease between the bonding layer of the PWAS and the plate and one with Teflon tape beneath the bonding layer of the PWAS and the plate. In instrumenting the defective specimen, grease and tape were placed between the plate and bonding agent for the PWASs, and the broken PWAS was damaged after being bonded to the specimen. The distance from each outer PWAS of this specimen to the inner pristine PWAS was 50 mm, equivalent to the distance between PWAS 3 and PWAS 4 in Figure 2. Figure 5 depicts the specimen instrumented with defective PWAS. The broken PWAS had a small chip in the top right of the sensor, referenced by the arrow in the figure. Each defective PWAS was used as both a transmitter and a receiver in experimentation, with the pitch-catch results compared with those of a pristine PWAS.

**Figure 6:**
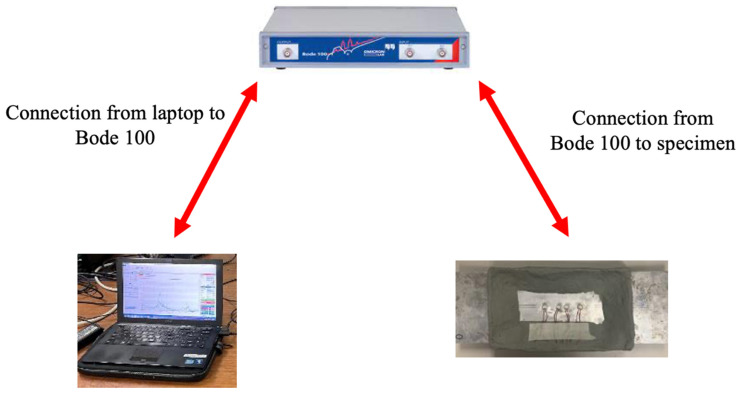
Experimental setup for conducting EMIS testing.

For this second specimen instrumented with defective instrumentation, EMIS testing was also performed. The experimental setup for the monitoring of PWAS bond quality by means of the EMIS is shown in Figure 6. In this experimental setup, a laptop was connected to the BODE 100 analyzer which communicates the test parameters. The specimen being tested was then connected to the analyzer and, while the test is being ran, the results are displayed to the laptop.

## 3. Experimental Results: Discussion of Results Obtained

**Figure 7:**
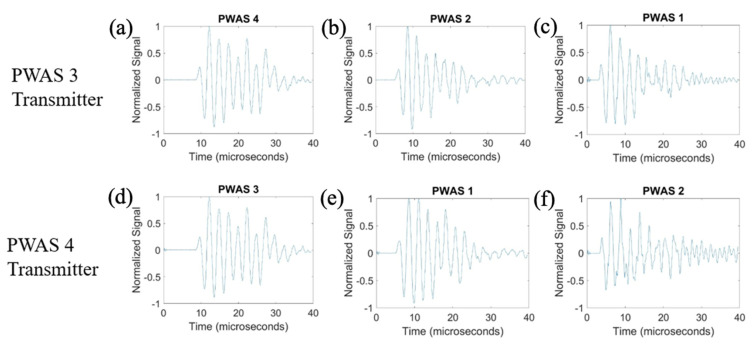
Comparison of received signals at (**a**) PWAS 4, (**b**) PWAS 2 and (**c**) PWAS 1 when PWAS 3 is actuating a smooth−delta function and (**d**) PWAS 3, (**e**) PWAS 1 and (**f**) PWAS 2 when PWAS 4 is actuating with a smooth−delta function.

**Figure 8:**
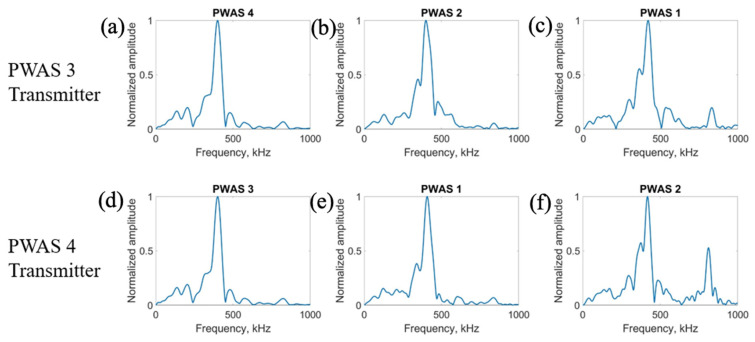
Comparison of frequency spectrums of received signals at (**a**) PWAS 4, (**b**) PWAS 2 and (**c**) PWAS 1 when PWAS 3 is actuating a smooth−delta function and (**d**) PWAS 3, (**e**) PWAS 1 and (**f**) PWAS 2 when PWAS 4 is actuating with a smooth−delta function.

On a specimen instrumented with four pristine PWASs, as pictured in Figure 2, the first excitation function used in pitch-catch experimentation is the smooth-delta function. Results for the comparison of the normalized signal received at each PWAS when PWASs 3 and 4 are the transmitting sensors are displayed in Figure 7. To account for slight variations in the EMISs of each PWAS, normalized signals are used for comparison. This is because the amplitude of signals may differ slightly between sensors due to their unique resonant frequencies when compared to the frequencies of the signal received. Normalizing the signal creates a uniform basis for comparing each signal received across all PWASs. When PWAS 3 is transmitting, the signals received at PWASs 4, 2 and 1 should be nearly identical to the signals received at PWASs 3, 1 and 2 when PWAS 4 is the actuating PWAS. By performing a fast Fourier transform of the signal received at each PWAS, the frequency spectrum of each signal is obtained. Comparisons of the frequency spectrum of signals received for when PWAS 3 and PWAS 4 are actuating, corresponding to the normalized signals in Figure 7, are shown in Figure 8.

From Figure 7, it can be seen that signals between PWAS 3 and PWAS 4 when the opposite is transmitting are nearly identical, while signals between PWAS 1 and 2 are not. The best comparison of the signals comes from analyzing the frequency spectrums, as differences in the signals become more apparent. From Figure 8, the frequency spectrums of the received signals at PWAS 3 and PWAS 4 are nearly identical when the opposite is actuating, as was the expected result with pristine instrumentation. It can be established that due to the nearly identical normalized signals received as well as the frequency spectrums of these signals, the instrumentation quality of both PWAS 3 and PWAS 4 are of matching and good quality. The frequency spectrums of PWAS 1 and PWAS 2 do not have this same identicality such that primary peak frequencies occur at similar values, but the secondary frequency peaks differ in their normalized amplitude as well as their values. The reasons for this disagreement in both the normalized signals received at PWAS 1 and 2, as well as the frequency spectrums of the signals, will be discussed later.

**Figure 9:**
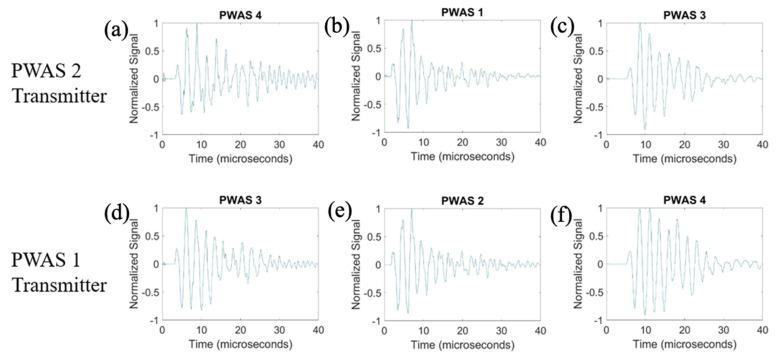
Comparison of received signals at (**a**) PWAS 4, (**b**) PWAS 1 and (**c**) PWAS 3 when PWAS 2 is actuating a smooth−delta function and (**d**) PWAS 3, (**e**) PWAS 2 and (**f**) PWAS 4 when PWAS 1 is actuating with a smooth−delta function.

**Figure 10:**
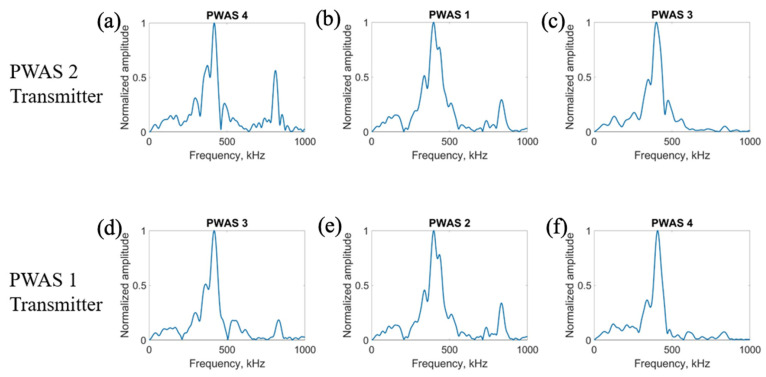
Comparison of frequency spectrums of received signals at (**a**) PWAS 4, (**b**) PWAS 1 and (**c**) PWAS 3 when PWAS 2 is actuating a smooth−delta function and (**d**) PWAS 3, (**e**) PWAS 2 and (**f**) PWAS 4 when PWAS 1 is actuating with a smooth−delta function.

The results for transmitting a delta function with PWASs 1 and 2 are displayed in Figure 9. The frequency spectrums of the signals received when PWASs 1 and 2 are transmitting are shown in Figure 10.

When PWAS 2 is transmitting, the signals received at PWASs 4, 1 and 3 should be nearly identical to the signals received at PWASs 3, 2 and 4 when PWAS 1 is transmitting. From Figure 9, the normalized signals received at PWASs 1 and 2 when the other is actuating are nearly identical to each other, which is the expected result with defect-less instrumentation quality. This identicality is again better seen in the frequency spectrums. For this actuation, there are differences in the signals received at PWASs 3 and 4. Depicted in Figure 10, the frequency spectrums for signals received at PWASs 1 and 2 are nearly identical to each other, with frequency peaks of the same normalized amplitude occurring at the same frequencies. However, for PWASs 3 and 4, the frequency spectrums are not identical. The frequency spectrums of PWASs 3 and 4 have peak frequencies at similar values but the similarity between the signals received ends there, with different secondary peaks as well as high frequency peaks.

**Figure 11:**
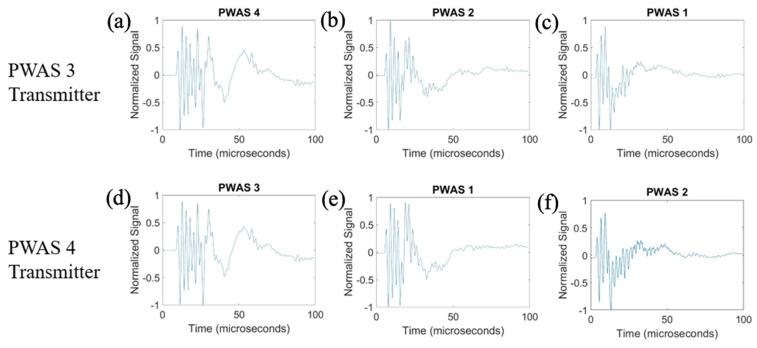
Comparison of received signals at (**a**) PWAS 4, (**b**) PWAS 2 and (**c**) PWAS 1 when PWAS 3 is actuating a smooth−step function and (**d**) PWAS 3, (**e**) PWAS 1 and (**f**) PWAS 2 when PWAS 4 is actuating with a smooth−step function.

**Figure 12:**
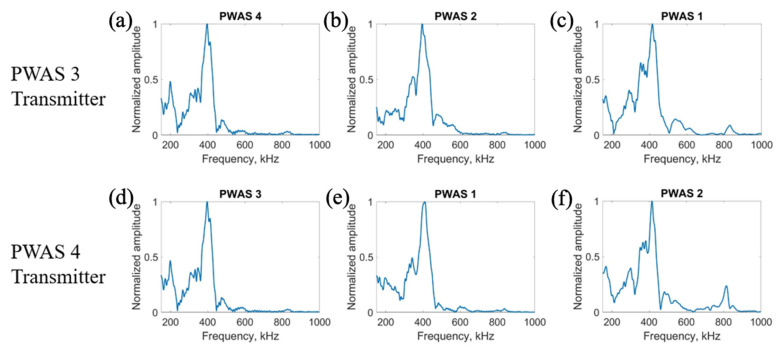
Comparison of frequency spectrums of received signals at (**a**) PWAS 4, (**b**) PWAS 2 and (**c**) PWAS 1 when PWAS 3 is actuating a smooth−step function and (**d**) PWAS 3, (**e**) PWAS 1 and (**f**) PWAS 2 when PWAS 4 is actuating with a smooth−step function.

After actuation with the delta function, the pristine specimen is then tested using the smooth-step function as the excitation function. Beginning with the actuation for PWASs 3 and 4, the results for normalized signals are displayed in Figure 11 and, by performing a fast Fourier transform of the signals, the frequency spectrums are obtained and pictured in Figure 12. It is again expected that when PWAS 3 is actuating, the signals received at PWASs 4, 2 and 1 should be nearly identical to the signals received at PWASs 3, 1 and 2 when PWAS 4 is actuating.

The received signals at PWASs 3 and 4 are nearly identical to each other when the other is actuating, which was to be expected with defect-less instrumentation quality. Between PWAS 1 and PWAS 2, the signals received are not identical to each other. The waveforms of PWASs 1 and 2 have similar shapes but differ in some oscillatory components in the signal. From Figure 12, the frequency spectrums of the signals received at PWAS 3 and PWAS 4 are identical to each other, with frequency peaks at nearly the same values and normalized amplitudes, while the frequency spectrums of the signals received at PWAS 1 and PWAS 2 are not. The signals between PWAS 1 and PWAS 2 have similar peak frequency values, but differences occur in the values of the secondary peaks and their normalized amplitudes. Again, we can establish that, due to the nearly identical normalized signals received and the frequency spectrum of these received signals, the instrumentation quality of both PWAS 3 and PWAS 4 are of matching and good quality.

**Figure 13:**
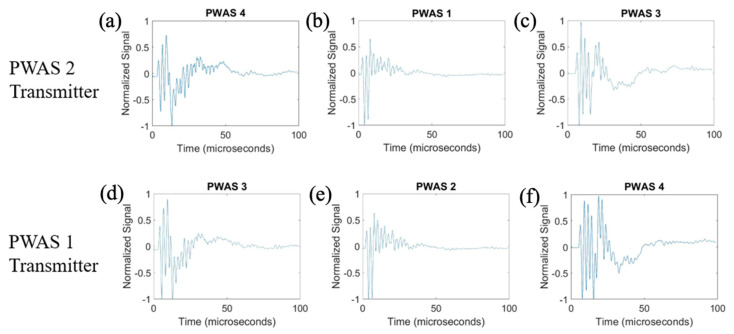
Comparison of received signals at (**a**) PWAS 4, (**b**) PWAS 1 and (**c**) PWAS 3 when PWAS 2 is actuating a smooth−step function and (**d**) PWAS 3, (**e**) PWAS 2 and (**f**) PWAS 4 when PWAS 1 is actuating with a smooth−step function.

**Figure 14:**
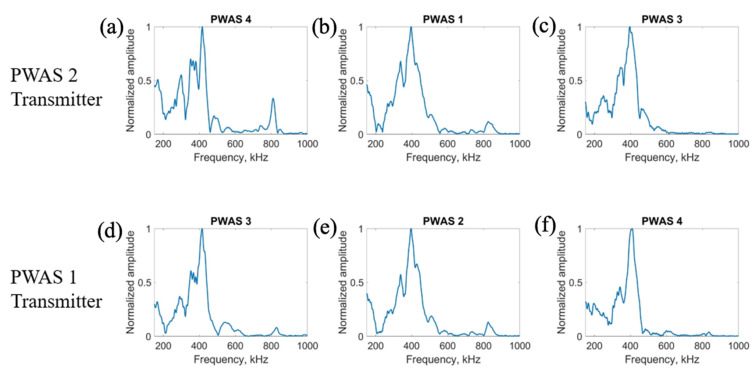
Comparison of frequency spectrums of received signals at (**a**) PWAS 4, (**b**) PWAS 1 and (**c**) PWAS 3 when PWAS 2 is actuating a smooth−step function and (**d**) PWAS 3, (**e**) PWAS 2 and (**f**) PWAS 4 when PWAS 1 is actuating with a smooth−step function.

Moving to the actuation of PWAS 1 and PWAS 2, it is again expected that when PWAS 2 is actuating, the signals received at PWASs 4, 1 and 3 should be nearly identical to the signals received at PWASs 3, 2 and 4 when PWAS 1 is actuating. The comparison of the normalized received signals is shown in Figure 13 and the comparison of the frequency spectrums of these received signals is pictured in Figure 14.

The signals received at PWAS 1 and PWAS 2 are again nearly identical to each other, while the other signals received at both PWAS 3 and PWAS 4 are not. The frequency spectrums for PWAS 1 and PWAS 2 are identical to each other, with frequency peaks and amplitudes of the same values, while the frequency spectrums for PWASs 3 and 4 are not identical to each other, with differing secondary frequency peaks and their normalized amplitudes. Again, due to the nearly identical normalized signals received and the frequency spectrums of these received signals, we can confirm that the instrumentation quality of both PWAS 1 and PWAS 2 are of matching and good quality.

In determination of PWAS instrumentation quality for this pristine specimen, it is assumed that PWAS instrumentation quality was defect-less. When actuating PWAS 3 and PWAS 4 with both the delta function and smooth-step function, the signals received at the other were identical to each other; thus, it can be understood that the instrumentation quality between both PWAS 3 and PWAS 4 is similar and defect-less. The same can be said for both PWAS 1 and PWAS 2, as when the opposite was the transmitting PWAS, the signals received at each sensor were identical, as were their frequency spectrums.

**Figure 15:**
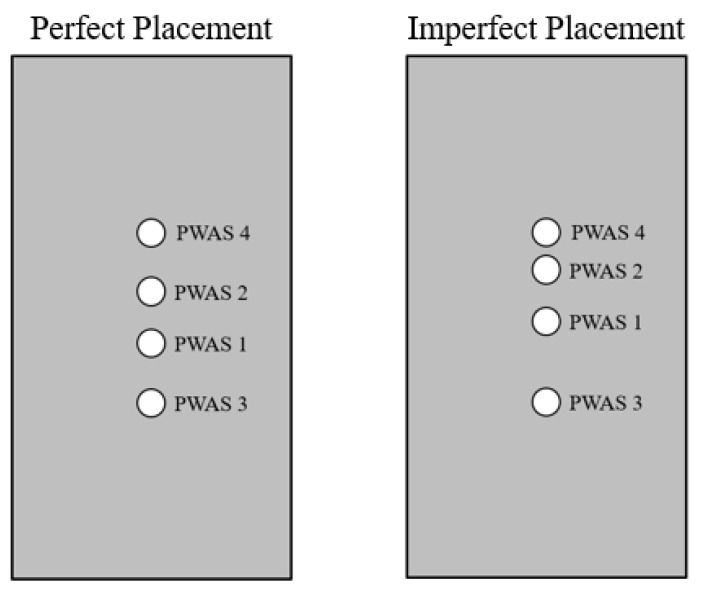
Comparison of perfect and imperfect placement of PWAS on specimen.

Explanations behind the result that some PWASs do not receive identical waveforms can be reduced to human error in the placement of the sensor. Slight differences in the distances between sensors due to their placement results in differences in the signals received based on travel distance of the signal. Seen in Figure 15, two fixed distances in placement of the sensors are the distance between PWAS 1 and PWAS 2 and the distance between PWASs 3 and 4, or the distance between the two inner and two outer PWASs. Regardless of the placement of the PWASs, the distance between these PWASs is fixed and does not change based on which PWAS is actuating. Because of this, signal comparison between these sensors should result in near-identical signals if the instrumentation of the specimen is defect-less. Comparisons of signals between other PWASs has the potential for mismatching signals due to differing distances between transmitting and receiving PWASs. Figure 15 depicts the difference between imperfect and perfect placement. The differences in distances in PWASs that should be symmetrical can be seen in the imperfect placement graphic, such as the differences in distances between PWASs 1 and 3 and PWASs 1 and 4. In this experiment, where the focus was identifying variations between nominally identical sensors, the distinct primary and secondary peaks were maintained across the experimental results and the similarly shaped frequency spectrums observed. This proved that nominally identical sensors give consistent results, though slight human error in placement may lead to slight changes in the spectrum.

**Figure 16:**
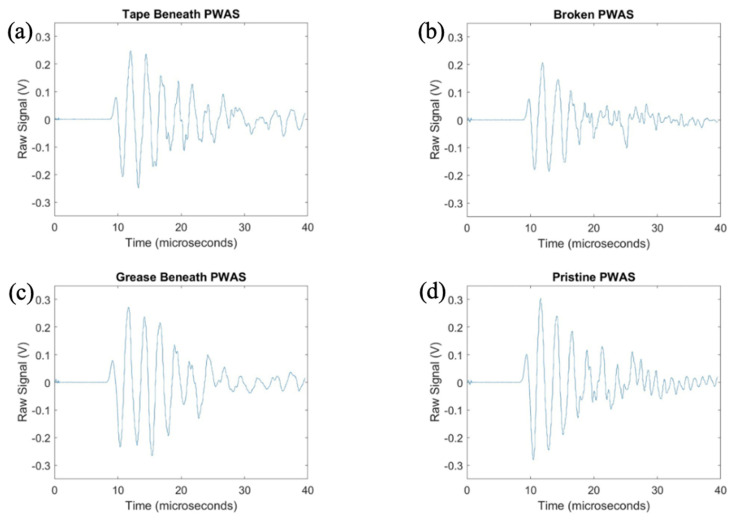
Comparison of delta function excitation signals received at (**a**) PWAS with tape beneath bonding layer, (**b**) a broken PWAS, (**c**) PWAS with grease beneath bonding layer and (**d**) a pristine PWAS.

**Figure 17:**
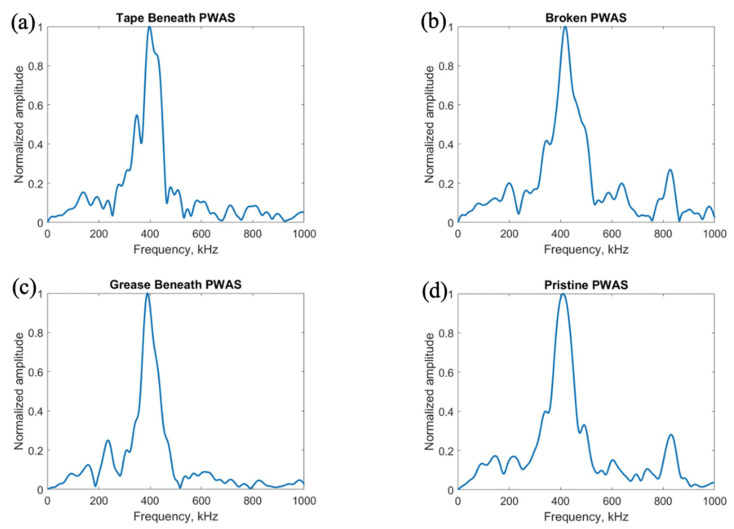
Comparison of the frequency spectrum of delta function excitation signals received at (**a**) PWAS with tape beneath bonding layer, (**b**) a broken PWAS, (**c**) PWAS with grease beneath bonding layer and (**d**) a pristine PWAS.

After exploring the use of the pitch-catch method for the determination of PWAS instrumentation quality, pitch-catch testing was performed on a specimen with defective instrumentation, pictured in Figure 5, to investigate the effects of defects on the PWAS response. When performing pitch-catch experiments for this specimen, each of the outer defective PWASs were analyzed in a pair with the inner pristine PWAS. The effects of defective instrumentation were analyzed, with defects occurring in both the transmitting and receiving PWASs. In this case, opposed to the normalized signal in previous experiments, the raw signal was analyzed to investigate the effect of defects in piezoelectric sensor instrumentation on the amplitude of the PWAS response. The same experimental setup was used, and the same smooth-step and smooth-delta functions shown previously in Figure 3 were used in the excitation of the sensors. In Figure 16, waveforms received at the outer PWASs are compared when the center pristine PWAS is transmitting a smooth-delta function. As compared to waveforms received at a pristine PWAS, all piezoelectric sensor instrumentation defects resulted in a decrease in the amplitude of the signal received. A broken PWAS experiences a greater decrease in signal amplitude compared to that of a PWAS with tape beneath and a PWAS with grease beneath the bonding layer. A comparison of the frequency spectrum of these signals is shown in Figure 17. The bonding layer defects of tape and grease between the plate and sensor bond result in the elimination of the high frequency peaks occurring at around 850 kHz in the frequency spectrum of the signals. Signals received at a broken PWAS retain the same peaks as those of signals received by a pristine PWAS. Differences in the frequency spectrum of broken and pristine PWASs occur at around 600 kHz, where the spectrum of a broken PWAS has increased amplitude compared to a pristine PWAS.

**Figure 18:**
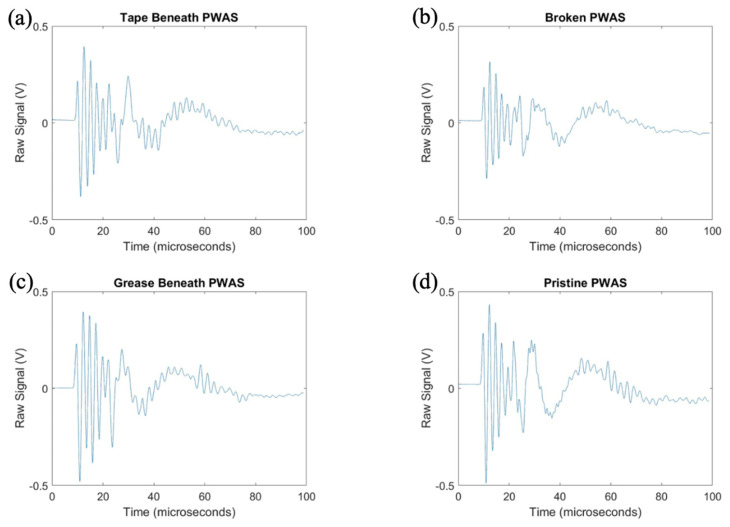
Comparison of step function excitation signals received (**a**) PWAS with tape beneath bonding layer, (**b**) a broken PWAS, (**c**) PWAS with grease beneath bonding layer and (**d**) a pristine PWAS.

**Figure 19:**
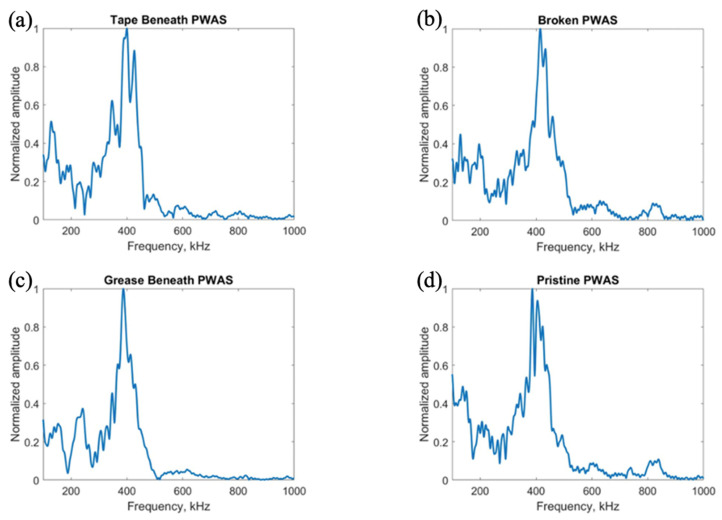
Comparison of the frequency spectrum of step function excitation signals received at (**a**) PWAS with tape beneath bonding layer, (**b**) a broken PWAS, (**c**) PWAS with grease beneath bonding layer and (**d**) a pristine PWAS.

After delta function excitation, the central PWAS was then excited using the smooth-step function, with comparison of the received signals shown in Figure 18. Similar to results obtained for delta function excitation, compared to a pristine PWAS, all PWAS instrumentation defects result in a decrease in the amplitude of the signal received. A broken PWAS continues to experience the greatest decrease in signal amplitude, while grease and tape bonding layer defects result in only a minor decrease in amplitude. The frequency spectrums of the signals are compared in Figure 19.

Like the frequency spectrums of signals received for delta function excitation, PWASs with defects in the bonding layer have similar peak frequencies but lack the secondary high frequency peak in the signal received by a pristine PWAS around 850 kHz. The signal received by a broken PWAS contains the same peak and secondary frequencies, but experiences increased amplitude of the frequency spectrum at around 600 kHz, similar to the case of delta function excitation.

**Figure 20:**
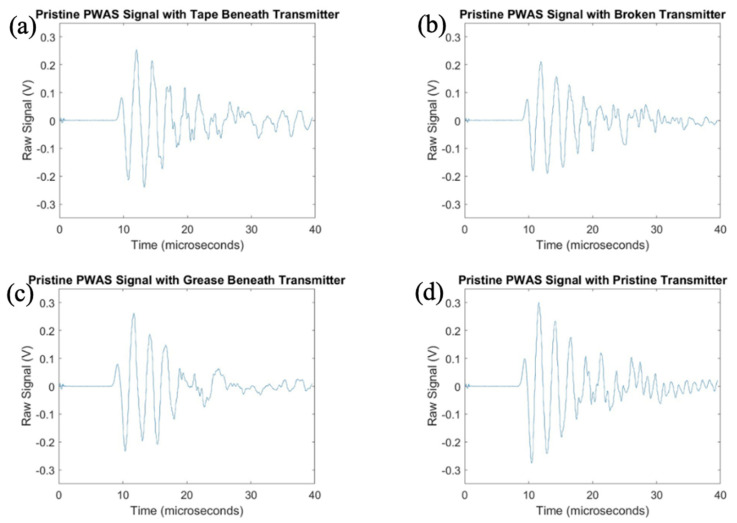
Comparison of delta function excitation signals received at a pristine PWAS with transmitting signals from (**a**) PWAS with tape beneath bonding layer, (**b**) a broken PWAS, (**c**) PWAS with grease beneath bonding layer and (**d**) a pristine PWAS.

**Figure 21:**
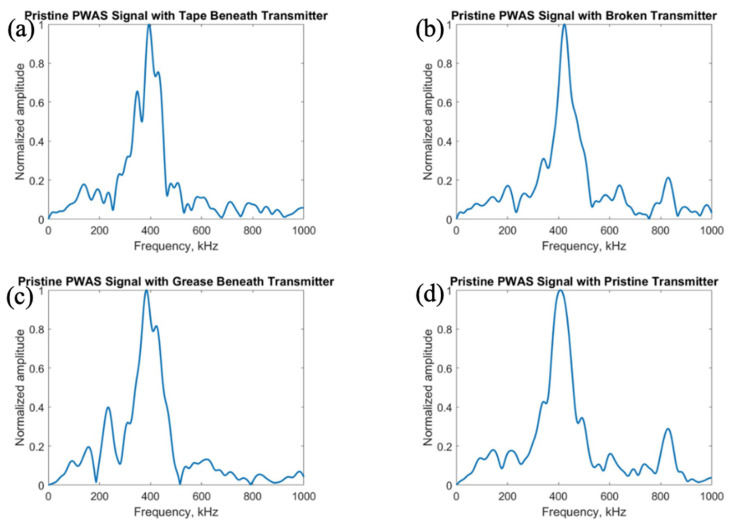
Comparison of frequency spectrums of delta function excitation signals received at a pristine PWAS with transmitting signals from (**a**) PWAS with tape beneath bonding layer, (**b**) a broken PWAS, (**c**) PWAS with grease beneath bonding layer and (**d**) a pristine PWAS.

After investigation of the effects of PWAS instrumentation defects on receiving PWAS during pitch-catch testing, the experimentation was flipped, and the signal transmitted by defective sensors was analyzed. The pristine center PWAS was used as the receiver and the outer defective PWAS used as the transmitting sensors. The signals received at the center pristine PWAS when each of the four outer PWASs are transmitting a delta function are shown in Figure 20, and the frequency spectrums are shown in Figure 21.

**Figure 22:**
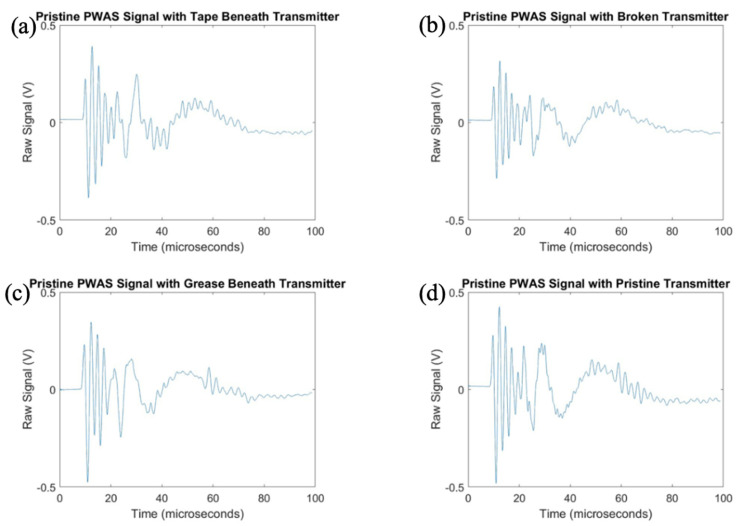
Comparison of step function excitation signals received at a pristine PWAS with transmitting signals from (**a**) PWAS with tape beneath bonding layer, (**b**) a broken PWAS, (**c**) PWAS with grease beneath bonding layer and (**d**) a pristine PWAS.

The signals transmitted by defective PWAS also have a decreased amplitude compared to a pristine transmitting PWAS. The greatest decrease in signal amplitude occurs with a broken PWAS, as both tape and grease beneath the transmitting sensor reduce the amplitude of the signal but not to the extent of a broken PWAS. Once again, the secondary frequency peak around 850 kHz has been eliminated in the signals transmitted from PWASs with tape and grease beneath the sensor bond. The broken PWAS contains similar primary and secondary frequency peaks as a pristine PWAS but also contains an increased amplitude in the spectrum at around 600 kHz. The signals received at the pristine inner PWAS, when each outer PWAS is transmitting a step function is shown in Figure 22.

**Figure 23:**
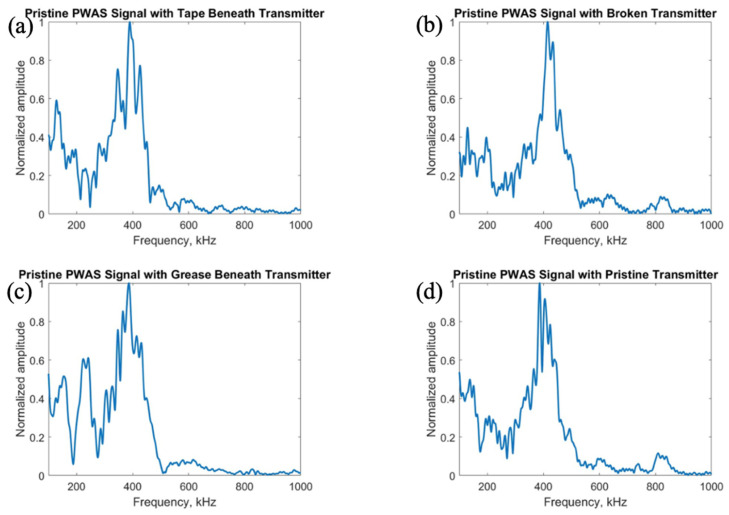
Comparison of frequency spectrums of step function excitation signals received at a pristine PWAS with transmitting signals from (**a**) PWAS with tape beneath bonding layer, (**b**) a broken PWAS, (**c**) PWAS with grease beneath bonding layer and (**d**) a pristine PWAS.

Consistent with previous results, the broken PWAS has had the greatest decrease in amplitude in comparison, and the sensors with tape and grease beneath the transducer also continually have decreased amplitude signals compared to a pristine PWAS but not to the degree of a broken sensor. Frequency spectrums for these signals are compared in Figure 23. Differences in frequency spectrums for PWASs with tape and grease beneath the bonding layer continue to include the lack of a secondary frequency peak around 850 kHz, as well as an increase in the amplitude of the frequency spectrum around 600 kHz for a broken PWAS.

An interesting note is the symmetry of signals from both a defective transmitter and a pristine receiver and a defective receiver with a pristine transmitter. Both a defective transmitter and receiver produce some of the same variations in the signal received. Explanations for this symmetry include that the PWAS defects affect the resonant frequencies of the sensor regardless of whether it is a transmitter or receiver, resulting in the same variation regardless of mode.

In this experiment, which focused on detecting the effect of sensor defects, the high frequency peaks were observed to collapse as a result of defective bonding, with the peak even disappearing entirely. Increased amplitude around 600 kHz in the frequency spectrum is considered to be a result of what appears to be an additional peak in the spectrum. This provides ways to identify defective sensor installation in practical applications.

**Figure 24:**
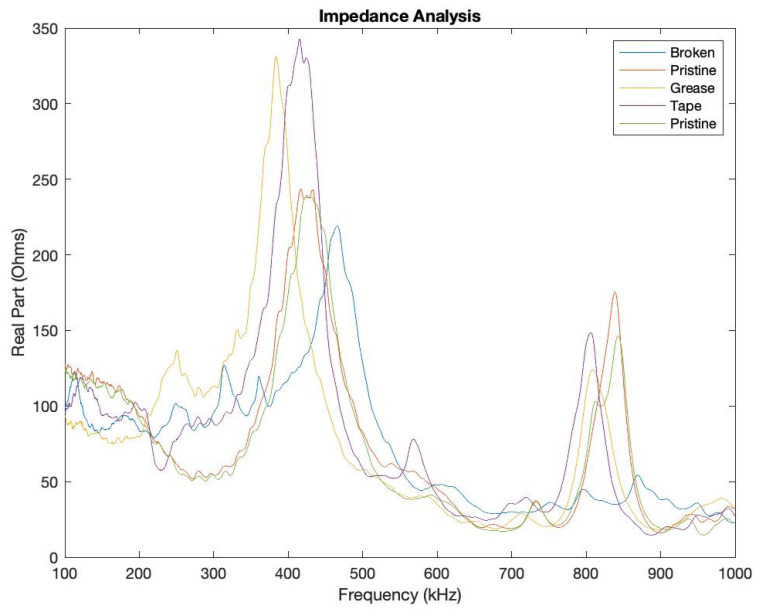
Complete EMIS for pristine, broken and defectively bonded PWAS.

**Figure 25:**
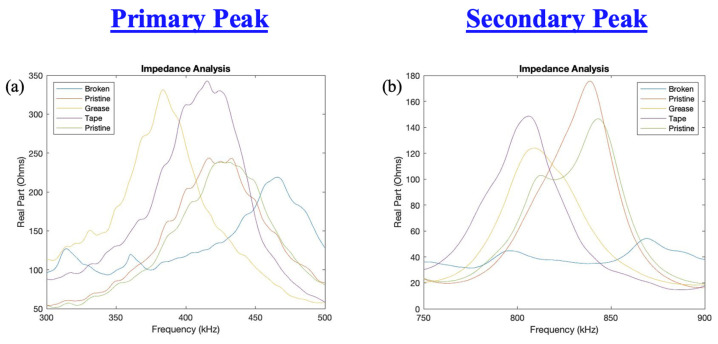
(**a**) Primary and (**b**) secondary peaks in the frequency spectrum for pristine, broken and defectively bonded PWAS.

EMIS testing was also performed on the specimen with defective instrumentation. In this instance, the real part of the impedance spectrum was analyzed. Results for testing are shown in Figure 24. Figure 25 provides an enhanced view of the primary and secondary frequency peaks. From these figures, it is seen that defects in bonding, in this case with grease and tape beneath the bond, result in an increased amplitude of the primary peak of the spectrum. This increased amplitude is accompanied by a downward shift in the frequency peak compared to a pristine PWAS. With bonding defects, the secondary peak maintains a similar amplitude but also experiences a downward shift in its frequency. A broken PWAS leads to a decrease in amplitude, as well as an upward shift of the primary frequency peak with the secondary peak becoming nearly completely eliminated. The EMIS testing confirms the instrumentation defects and gives confidence in the results obtained.

## 4. Summary and Conclusions

### 4.1. Summary

SHM is of great importance because it allows for design feedback, performance enhancement, on-demand condition-based maintenance and predictive fleet-level prognosis. PWASs have proven to be useful in SHM, with their use in the active sensing of far-field damage using pulse-echo, pitch-catch and phased array methods, active sensing of near-field damage using the electromechanical impedance method and passive sensing of acoustic emissions from crack advancement in addition to impact detection. The bonding and quality of sensors used in SHM is of particular importance as defective bonding or sensors can produce misleading measurements, signaling damage where there is not or resulting in the inability to detect structural damage. Many of the current methods used to monitor piezoelectric sensor quality center around the electromechanical impedance method and tracking changes in the impedance spectrum of the sensor and bonded structure.

A new method for the determination of piezoelectric bond and sensor quality has been developed to be used in the verification of instrumentation quality for sensors used in the collection of acoustic emissions. This method utilizes the pitch-catch of lamb waves through piezoelectric actuation and sensing to verify that each sensor and its bond to the structure is defect-less. Functions used in actuation of sensors in this method include a smooth-step function, which simulates an acoustic emission occurring from fatigue crack growth, and a smooth-delta function, which simulates fatigue crack rubbing and clapping.

An investigation into the effects of defects in PWAS instrumentation on the sensor response during pitch-catch experimentation was also conducted. A specimen was instrumented with five PWASs, two pristine, one with grease beneath the sensor bond, one with Teflon tape beneath the sensor bond, and one broken PWAS. Pitch-catch testing was performed within pairs of PWASs, one with a defect and one pristine PWAS. Each defective PWAS was used as both a transmitter and receiver in the experimentation and the signals received and transmitted were compared. EMIS testing was also performed on this specimen with defective instrumentation.

### 4.2. Conclusions

Existing approaches for the assessment of PWAS instrumentation quality largely concentrate on the evaluation of resonant frequencies of a sensor as opposed to directly measuring the sensing and receiving capabilities. However, a pitch-catch method allows for the analysis of the sensor quality as well as the impact of sensor defects on an acoustic emission signal. This pitch-catch method for determination of PWAS instrumentation quality was proven to be capable of analyzing and verifying defect-less piezoelectric sensor instrumentation on a pristine instrumented specimen, with some limitations. When using this method, although PWAS placement was assumed to be perfectly symmetric, in reality this is not the case as they are prepared by hand, introducing human error in placement of the sensors. For this reason, when examining piezoelectric sensors in this method, they should only be analyzed in pairs of sensors, as the distance between a “pair” of sensors is constant, whereas when trying to analyze multiple PWASs or groups of PWASs at a time, there will be slight differences in distances between PWASs, resulting in variations in signals and signal comparison.

When excited as a transmitter and when used as a receiver, a broken PWAS experienced the greatest decrease in signal amplitude. PWASs with bonding defects experienced only a minor decrease in signal amplitude when used as both the transmitting and receiving PWAS. Symmetry between signals was observed between defects occurring in both the actuating and receiving PWASs, in that they both produced the same variation in signal. Effects of defective bonding on the PWAS signal included the elimination of the high frequency peak around 850 kHz when both the delta and step function are used. A broken piezoelectric sensor results in increased amplitude of the frequency spectrum around 600 kHz. Broadly speaking, PWAS instrumentation defects result in a decrease in amplitude of the signal received, as well as a change in the frequency spectrum of the signal regardless of whether the defect is in the transmitting or receiving sensor. EMIS testing on defective sensor instrumentation gave confidence in the pitch-catch results obtained, while concluding that defects in the bonding layer of the sensor increase the primary frequency peak’s amplitude and cause a downward frequency shift. The secondary peak also experiences a downward shift but maintains similar amplitude. The EMIS of a broken sensor has a decreased amplitude while shifting the primary peak upward and nearly eliminating the secondary peak.

## Data Availability

Not applicable.
